# Fragility of a multilayer network of intranational supply chains

**DOI:** 10.1007/s41109-020-00310-1

**Published:** 2020-09-23

**Authors:** Michael Gomez, Susana Garcia, Sarah Rajtmajer, Caitlin Grady, Alfonso Mejia

**Affiliations:** 1 Department of Civil and Environmental Engineering, The Pennsylvania State University, State College, USA; 2 Energy and Transportation Science Division, Oak Ridge National Laboratory, Oak Ridge, USA; 3 College of Information Sciences and Technology, The Pennsylvania State University, State College, USA; 4 The Rock Ethics Institute, The Pennsylvania State University, State College, USA

**Keywords:** Economic networks, COVID-19, Shock, Cascading failure, Multiplex, Diffusion, Multiregional input-output

## Abstract

Supply chains enable the flow of goods and services within economic systems. When mapped for the entire economy and geographic locations of a country, supply chains form a spatial web of interactions among suppliers and buyers. One way to characterize supply chains is through multiregional input-output linkages. Using a multiregional input-output dataset, we build the multilayer network of supply chains in the United States. Together with a network cascade model, the multilayer network is used to explore the propagation of economic shocks along intranational supply chains. We find that the effect of economic shocks, measured using the avalanche size or total number of collapsed nodes, varies widely depending on the geographic location and economic sector of origin of a shock. The response of the supply chains to shocks reveals a threshold-like behavior. Below a certain failure or fragility level, the avalanche size increases relatively quickly for any node in the network. Based on this result, we find that the most fragile regions tend to be located in the central United States, which are regions that tend to specialize in food production and manufacturing. The most fragile layers are chemical and pharmaceutical products, services and food-related products, which are all sectors that have been disrupted by the Coronavirus Disease 2019 (COVID-19) pandemic in the United States. The fragility risk, measured by the intersection of the fragility level of a node and its exposure to shocks, varies across regions and sectors. This suggests that interventions aiming to make the supply-chain network more robust to shocks are likely needed at multiple levels of network aggregation.

## Introduction

In economic systems, a shock that originates in a single economic sector or geographic location can propagate to affect across many different sectors and locations (Bak et al. 1993; Delli Gatti et al. 2009; May et al. 2008; Schweitzer et al. 2009). Understanding the severity and mechanisms behind such cascading failures has become increasingly relevant as evidenced by recent catastrophic events, such as the global 2007–2008 financial crises (Acharya and Richardson 2009; May et al. 2008) and the Coronavirus Disease 2019 (COVID-19) pandemic (Bunge and Newman 2020; Jacobs et al. 2020). For example, the 2007–2008 financial crisis initially originated in the real-estate sector of the United States but ultimately resulted in a global economic recession (Acharya and Richardson 2009). To analyze and model the system-level repercussions of shocks, economic systems can be represented as complex networks (Acemoglu et al. 2016b; Schweitzer et al. 2009). Taking a system-level view is crucial in order to account for complex pathways and interdependencies among numerous economic agents spanning short- and long-range interaction scales (Lee and Goh 2016; Lee et al. 2011; Schweitzer et al. 2009; Vespignani 2010).

Several authors have shown that the underlying network structure of economic systems plays a fundamental role in the diffusion of economic shocks (Acemoglu et al. 2016b; Bak et al. 1993; Bardoscia et al. 2017; Haldane and May 2011; Lee et al. 2011). These findings, however, have been mostly based on the analysis of single-layer networks or interactions at the national or international level (Acemoglu et al. 2012; Contreras and Fagiolo 2014; Lee et al. 2011). Much less is known about the ability of shocks to lead to cascading failures in intranational economic networks. Here, using an empirical multilayer network of the flow of economic goods and services across geographic locations in the United States, together with a network diffusion model, our goal is to explore the transmission of economic shocks along intranational supply chains.

The need to better understand the response of intranational supply-chain networks to sudden changes in supply and demand has become evident with the global COVID-19 pandemic (Bunge and Newman 2020). The pandemic has exposed the fragility of supply chains in the United States (Bunge and Newman 2020; O’Leary 2020). For example, with the pandemic shortages of key medical supplies, drugs and various consumer staple products have been widespread across the country (Jacobs et al. 2020; Oremus 2020). Shortages have been attributed to disruptions in international imports, hoarding, just-in-time inventories, and lack of coordinated system-level actions (O’Leary 2020; Oremus 2020), among other factors. Despite shortages being likely driven by multiple factors, it is apparent that once a shock is triggered in a particular location it can spread broadly along supply chains to cause distress in other locations (Bunge and Newman 2020; Jacobs et al. 2020; O’Leary 2020; Oremus 2020). It is also apparent that more needs to be done to contain or mitigate the propagation of undesirable and damaging shocks (Whitehouse 2020). By exploring the transmission of shocks in supply chains of the United States, we seek to understand whether there are supply-chain sectors that are more fragile than others, and whether some regions are more shock prone than others. Such information could help strengthen supply chains where it is needed most.

## Background

Network science approaches have been used to analyze the interplay between structure and shock dynamics in various types of economic networks (Fagiolo 2016; Schweitzer et al. 2009), such as financial (Battiston et al. 2016; Delpini et al. 2019; Perillo and Battiston 2018; Starnini et al. 2019), firm (Hisano et al. 2017; Joyez 2017), trade (Bonaccorsi et al. 2019; Garlaschelli and Loffredo 2004; Serrano and Boguñá 2003), and supply-chain (Inoue and Todo 2019; Matous and Todo 2017; Perera et al. 2017; Piccardi et al. 2017) networks. Our focus in this study is on supply-chain networks as represented by economic input-output data. A number of studies have examined subnational supply-chain networks associated with a specific group of firms or sectors (Perera et al. 2017). In contrast, using input-output data, our goal is to account for interactions across many different economic sectors.

Previous studies have used input-output data to examine shock propagation at the national level (Acemoglu et al. 2016a; Blöchl et al. 2011; McNerney et al. 2013). In those studies, the nodes of the network are economic sectors, i.e. groups of firms that produce similar goods or services, and the links between the nodes are flows of goods and services. For instance, using a macroeconomic model and input-output data for the United States, Acemoglu et al. (2012) showed that sectoral-level idiosyncratic shocks can influence other sectors to generate aggregate (national-level) fluctuations. That is, sectoral-level shocks can lead to cascading failures that potentially result in economic downturns. They found that sectoral interdependencies serve as a mechanism that amplifies shocks at the national level. Likewise, other authors have used input-output data for different countries to explore various aspects of the topology and proneness of sectoral interdependencies to reveal cascading effects (Blöchl et al. 2011; Contreras and Fagiolo 2014; McNerney et al. 2013).

In this study, we use a multiregional input-output (MRIO) dataset to build the multilayer network of supply chains in the United States (Garcia et al. 2020a). MRIO accounts couple input-output data for different regions to represent the flow of economic goods and services across both economic sectors and geographic locations (Miller and Blair 2009). To capture all the different interaction types implied by MRIO data, such as economic flows across regions within the same sector (intralayer flows) and across different sectors (interlayer flows), it is necessary to represent MRIO data using a multilayer network (Aleta and Moreno 2019; Kivelä et al. 2014). The inclusion of interlayer flows or couplings is crucial as they have been shown to enhance the propagation of shocks (Vespignani 2010). Multilayer networks built using MRIO data have recently been used to analyze international economic flows among countries (Cerina et al. 2015; Cingolani et al. 2017; del Río-Chanona et al. 2017; Maluck and Donner 2015; Piccardi et al. 2017; Xing et al. 2017). This is to our knowledge the first application of multilayer networks to study shock propagation within intranational MRIO data.

Different approaches have been employed to model diffusion processes in multilayer networks (Aleta and Moreno 2019; Bianconi 2018; Boccaletti et al. 2014; Kivelä et al. 2014). Several authors have performed comprehensive reviews of these approaches (Bródka et al. 2020; de Arruda et al. 2018; De Domenico et al. 2016; Kivelä et al. 2014; Salehi et al. 2015), therefore we do not review them here. To model the propagation of shocks in our multilayer network of supply chains, we use a network cascade model (Buldyrev et al. 2010; Kinney et al. 2005; Lorenz et al. 2009; Niu et al. 2018). Cascade models have previously been shown to work well with economic input-output data (Contreras and Fagiolo 2014). We use a simulation-based approach because empirical data to characterize the propagation of shocks in supply chains are not readily available. The next section describes the data utilized to build the multilayer network and the cascade model. Following, we present and discuss the main results from the application of the model.

## Materials and methods

### Multiregional input-output dataset

To build the multilayer network of supply chains in the United States, we use a recently developed MRIO dataset (Garcia et al. 2020b). The dataset maps the domestic flows of economic goods and services across sectors and regions in the United States economy during 2012, which is the latest year with publicly available data to calibrate the MRIO dataset (Garcia et al. 2020b). The dataset covers the entire contiguous United States through 115 different geographic regions, including 65 major cities and 50 remainders of states or states. A remainder of state is the area of a state that is not part of one of the cities in the dataset. There are also a few states without any cities in them. These are treated as single geographic locations. The dataset accounts for 37 different types of flows or economic sectors (Table 1), including 8 food-related sectors, 23 industrial or manufacturing sectors, and 6 service sectors. Since these 37 sectors can be present in any of the regions in the dataset, the total number of sectors is 4255 (37 × 115).

**Table 1:** List of the 37 economic sectors in the multiregional input-output dataset

Economic Sectors			
Live animals	Building stones etc.	Wood products	Transport. equipment
Cereal grains	Nonmetallic minerals	Paper products	Precision instruments
Fruits and vegetables	Metallic ores	Printed products	Furniture
Animal feed	Basic chemicals	Textiles, leather	Utilities
Meat	Pharmaceuticals	Nonmetal minerals	Wholesale
Milled grain	Fertilizers	Metals^a^	Retail
Manufactured food	Chemical products	Machinery	Transport. services
Alcoholic beverages	Plastics and rubber	Electronics	Food services
Tobacco products	Logs	Vehicles	Government

^a^The metals sector includes two classes of commodities: raw metals and metal products

The MRIO dataset consists of outputs (production) for each of the 4255 sectors, inputs needed by each sector to produce those outputs (intermediate demand), and household consumption of the sectors’ outputs in each region (final demand). Using the basic input-output economic relationship (Isard 1951; Leontief et al. 1953), these data are related as

1 $$ \mathbf{x}=\mathbf{Z}\mathbf{1}+\mathbf{d}, $$

where $\mathbf{x}=\left\{{x}_i^{\alpha}\right\}\in {\mathrm{\mathbb{R}}}^{4255\times 1}$ with ${x}_i^{\alpha }$ being the output of sector *α* in region *i* [USD]. $\mathbf{Z}=\left\{{Z}_{ij}^{\alpha \beta}\right\}\in {\mathrm{\mathbb{R}}}^{4255\times 4255}$ with ${Z}_{ij}^{\alpha \beta}$ being the output of sector *α* in region *i* that is used as a production input by sector *β* in region *j* [USD], where 1 ≤ *i*, *j* ≤ 115 and 1 ≤ *α*, *β* ≤ 37. **1** is an appropriately scaled column vector of 1 s. $\mathbf{d}=\left\{{d}_i^{\alpha}\right\}\in {\mathrm{\mathbb{R}}}^{4255\times 1}$, where ${d}_i^{\alpha }$ is the final demand for products produced by sector *α* in region *i* [USD]. **Z** is used to represent the supply chains since it captures in a spatially explicit manner the inflows and outflows of goods and services needed for the economy to function. There is a more general way of expressing Eq. (1) to consider production and final demand flows across regions (Garcia et al. 2020b; Miller and Blair 2009). However, for the application of our network diffusion model, we only utilize the matrix **Z**. Thus, Eq. (1) captures all the information needed to build our multilayer network.

### Building the multilayer network

Using a graph representation (Alves et al. 2018, 2019), we define the multilayer network of supply chains as a pair *M* = (*G*, *C*). *G* is the family of directed graphs representing each of the layers (economic sectors), such that *G* = {*G*_*α*_; *α* ∈ {1, …, 37}} where *G*_*α*_ = {*V*_*α*_, *E*_*α*_}. For a layer *α*, *V*_*α*_ is the set of nodes (regions) and *E*_*α*_ is the set of directed edges between pairs of nodes (intralayer links) within the same layer. Every layer shares the same number of nodes that correspond to our 115 geographic locations. *C* is defined as

2 $$ C=\left\{{E}_{\alpha \beta}\subseteq {V}_{\alpha}\times {V}_{\beta };\alpha, \beta \in \left\{1,\dots, 37\right\},\alpha \ne \beta \right\}. $$

*C* is the set of couplings or interconnections (interlayer links) between pair of nodes located in different layers *G*_*α*_ and *G*_*β*_ with *α* ≠ *β*.

**Fig. 1:**
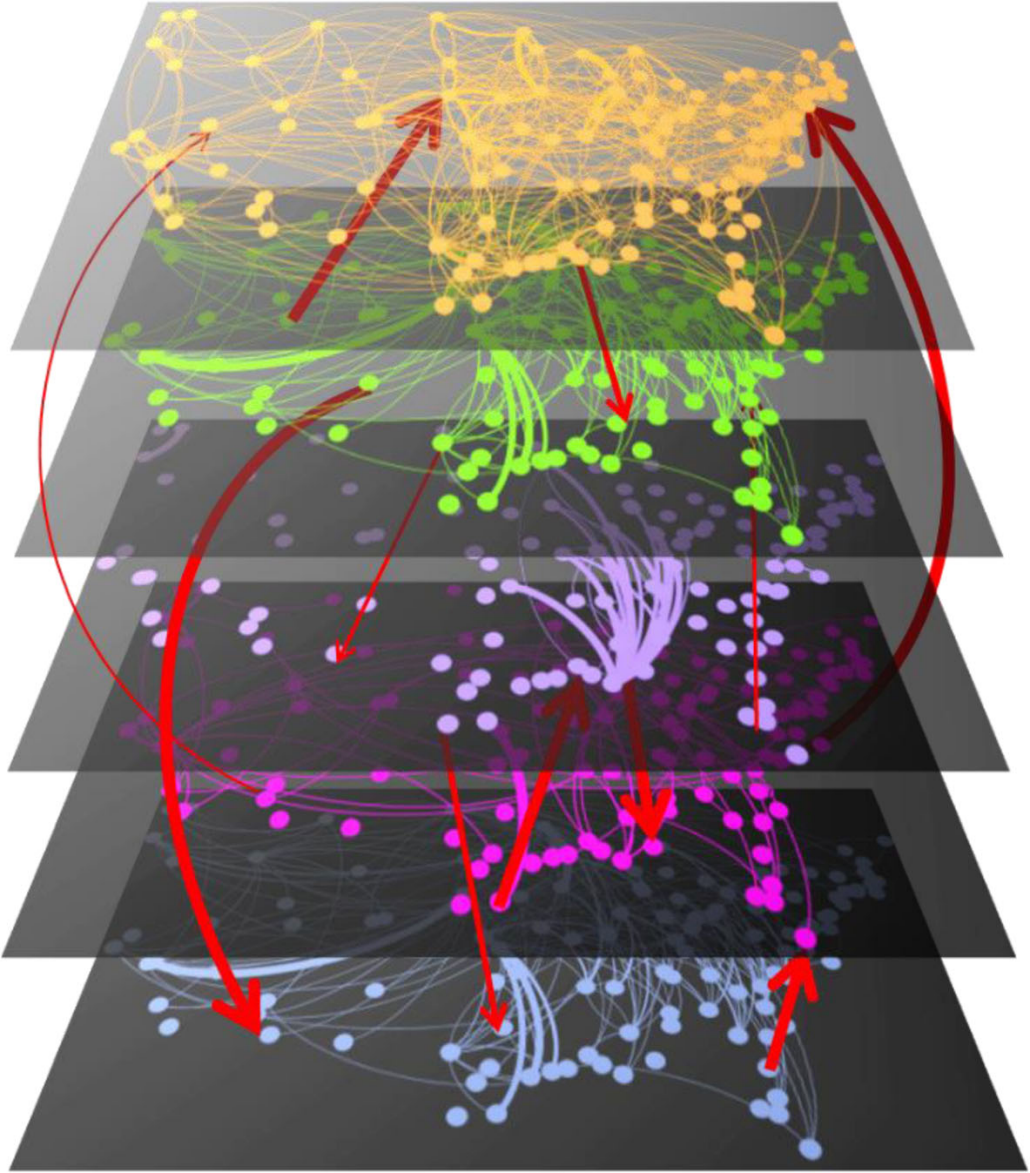
Illustration of the multilayer network of supply chains in the United States obtained using the multiregional input-output dataset. The illustration shows five different layers obtained from the actual data (intralayer flows) and a few hypothetical interlayer flows (red arrows). The nodes in each layer represent the 115 geographic locations or regions in the dataset. The same set of nodes are shared by all the layers

We use the MRIO matrix **Z** to identify the intralayer and interlayer links, and assign weights to the links (Fig. 1). This is done by constructing the supra-adjacency matrix as follows

3 $$ {S}_{ij}^{\alpha \alpha}=\left\{\begin{array}{l}{Z}_{ij}^{\alpha \alpha}\ \mathrm{if}\kern0.5em \left(i,j\right)\in {E}_{\alpha}\\ {}0\kern1.25em \mathrm{otherwise},\end{array}\right. $$

where ${\mathbf{S}}_{\boldsymbol{\upalpha}}=\left\{{S}_{ij}^{\alpha \alpha}\right\}\in {\mathrm{\mathbb{R}}}^{4255\times 4255}$ is the intralayer adjacency matrix and ${S}_{ij}^{\alpha \alpha}$ is the intralayer weight between nodes (*i*,*α*) and (*j*,*α*). **S**_**α**_ represents the flow of goods and services within layers. The elements ${S}_{ij}^{\alpha \beta}$ of the interlayer adjacency matrix ${\mathbf{S}}_{\boldsymbol{\upalpha} \boldsymbol{\upbeta}}=\left\{{S}_{ij}^{\alpha \beta}\right\}\in {\mathrm{\mathbb{R}}}^{4255\times 4255}$ corresponding to the set of couplings *E*_*αβ*_ are defined as

4 $$ {S}_{ij}^{\alpha \beta}=\left\{\begin{array}{l}{Z}_{ij}^{\alpha \beta}\kern0.5em \mathrm{if}\ \left(i,j\right)\in {E}_{\alpha \beta}\\ {}0\kern1.5em \mathrm{otherwise},\end{array}\right. $$

where ${S}_{ij}^{\alpha \beta}$ is the interlayer weight between nodes (*i*,*α*) and (*j*,*β*). **S**_**αβ**_ represents the flow of goods and services across layers. The supra-adjacency matrix for the multilayer network is then equal to **S**_**α**_ + **S**_**αβ**_.

### Network cascade model

We use a network cascade model (Contreras and Fagiolo 2014; Kinney et al. 2005; Lee et al. 2011) to explore the propagation of shocks along the multilayer network of supply chains. Following Contreras and Fagiolo (2014), the cascade model simulates the propagation of a shock starting at a source node in the network and propagating to other nodes through sectoral interdependencies. Recall that a node in the multilayer network represents a specific geographic location within a layer or economic sector. It is assumed that a shock to the source node is exogenous and sufficiently large to collapse the source node (Bunge and Newman 2020; Inoue and Todo 2019; May et al. 2008). Once the source node or any other node in the network collapses, it stays in that state for the remainder of the cascade event (Kleinberg 2007).

Specifically, the propagation of a shock or cascade event is simulated as a discrete process, where the shock is sequentially diffused along the MRIO supply chains. During the cascade event, the failure state of node *i* in layer *α* at time step *t* is defined by the variable ${f}_i^{\alpha }(t)\in \left\{0,1\right\}$. The failure state is ${f}_i^{\alpha }(t)=1$ if the node fails and ${f}_i^{\alpha }(t)=0$ otherwise. The cascade state at time step *t* is characterized by the state vector $\mathbf{f}(t)=\left\{{f}_i^{\alpha }(t)\right\}\in {\mathrm{\mathbb{R}}}^{4255\times 1}$. Before the start of a cascade, the initial failure state is ${f}_i^{\alpha }(0)=0$ for all nodes.

The cascade process is initiated by setting the state of the source node (*s*, ζ) to ${f}_s^{\zeta }(1)=1$. Any node in the network can act as a source. Once the cascade begins, the weight of the edges connected to the source node are reduced by a fraction *c*, 0 < *c* < 1. This is equivalent to decreasing the supply and demand of goods and services for (*s*, ζ), therefore *c* sets the magnitude or severity of the shock. This also means that all the neighbors of (*s*,ζ) are affected by the shock. For any neighboring node (*p*,*μ*) of (*s*,ζ), (*p*, *μ*) ∈ *N*(*s*, *ζ*), if the total change in flows is greater than the threshold capacity of the node, the node collapses and its state is changed to ${f}_p^{\mu }(1)=1$. The threshold capacity of a node is assumed to be a fraction *b*, 0 < *b* < 1, of its total production. Thus, *b* determines the capacity of a neighboring node to buffer or resist the shock originating from the source node.

Mathematically, after an exogenous shock is applied to the source node (*s*,ζ), the weights associated with it are reduced by a fraction *c* such that ${S}_{sp}^{\zeta \mu \ast }=\left(1-c\right){S}_{sp}^{\zeta \mu}$ and ${S}_{ps}^{\mu \zeta \ast }=\left(1-c\right){S}_{ps}^{\mu \zeta}$. Hence, the change in strength $\Delta {S}_p^{\mu }$ of a neighbor (*p*,*μ*) of the source node is evaluated as

5 $$ \Delta {S}_p^{\mu }=\sum \limits_{\lambda}\sum \limits_q\left({S}_{pq}^{\mu \lambda}-{S}_{pq}^{\mu \lambda \ast}\right)+\sum \limits_{\lambda}\sum \limits_q\left({S}_{qp}^{\lambda \mu}-{S}_{qp}^{\lambda \mu \ast}\right), $$

where (*q*,*λ*) is any neighbor of (*p*,*μ*), (*q*, *λ*) ∈ *N*(*p*, *μ*). The shock propagates from the source node to any of its neighbors if the following condition is met

6 $$ \Delta {S}_p^{\mu }>{bx}_p^{\mu }. $$

The term ${bx}_p^{\mu }$ is the threshold capacity of node (*p*,*μ*) and ${x}_p^{\mu }$ is the node’s total production. The condition in Eq. (6) says that a neighboring node fails when the total change of its supply and demand for goods and services surpasses its buffering capacity to resist the shock.

At the next time step, the neighbors to the source node that failed at the initial time step decrease their inflows and outflows by a fraction *c* and the shock continues to propagate. The cascade continues until there are no more failed nodes during a time step, i.e. until the shock is fully buffered by nodes with sufficient capacity. At the end of the cascade process, after *T* time steps, the size of the avalanche caused by a shock that originated in the source node (*s*,ζ), ${V}_s^{\zeta }$, is calculated as the total fraction of failed nodes such that

7 $$ {V}_s^{\zeta }=\frac{1}{m}\sum \limits_{\alpha}\sum \limits_i\sum \limits_t{f}_i^{\alpha }(t), $$

where *m* is the total number of sectors, 4255. We use the avalanche size ${V}_s^{\zeta }$ to characterize the shock propagation process.

Furthermore, by noting that at most two weighted edges are altered as the shock propagates to a neighboring node (*p*,*μ*), while all the other weighted edges of (*p*,*μ*) stay the same, the condition in Eq. (5) can be simplified to

8 $$ \Delta {S}_p^{\mu }=\left({S}_{pq}^{\mu \lambda}-{S}_{pq}^{\mu \lambda \ast}\right)+\left({S}_{qp}^{\lambda \mu}-{S}_{qp}^{\lambda \mu \ast}\right). $$

Using ${S}_{pq}^{\mu \lambda \ast }=\left(1-c\right){S}_{pq}^{\mu \lambda}$ and ${S}_{qp}^{\lambda \mu \ast }=\left(1-c\right){S}_{qp}^{\lambda \mu}$, we can write Eq. (8) as

9 $$ \Delta {S}_p^{\mu }=c\left({S}_{pq}^{\mu \lambda}+{S}_{qp}^{\lambda \mu}\right). $$

Substituting into Eq. (6), the condition for the failure of a node is now expressed as

10 $$ \frac{\left({S}_{pq}^{\mu \lambda}+{S}_{qp}^{\lambda \mu}\right)}{x_p^{\mu }}>\Omega, $$

where Ω = *b*/*c* is the failure threshold. This condition says that a stronger interaction between nodes (*p*,*μ*) and (*q*,*λ*) relative to the total outflows (production) of (*p*,*μ*) increases the possibility of failure for (*p*,*μ*). This is the case because, under a strong interaction strength and fixed shock severity *c*, the buffering capacity *b* of (*p*,*μ*) needs to be larger for the node not to fail. Therefore, the left-hand-side term in Eq. (10) can be considered a measure of node fragility, or lack of robustness, with respect to the global failure threshold Ω (Lorenz et al. 2009).

The condition in Eq. (10) shows that the cascade model only depends on Ω. In general, the value of Ω determines one of three possible cascade regimes. For Ω > 1, the buffering capacity of a node is greater than the shock severity, *b* > *c*. For Ω = 1, the buffering capacity and shock severity are equal, *b* = *c*. For Ω < 1, the shock severity dominates the buffering capacity, *b* < *c*. We are interested in exploring the latter regime since it can lead to catastrophic failures or large avalanche sizes.

### Implementation of the network cascade model

We use Ω to analyze how the avalanche size ${V}_s^{\zeta }$ behaves under different combinations of the shock severity *c* and buffering capacity *b*. For this, we independently apply a shock to each node in the multilayer network using different values of Ω, ranging from 0.02 to 0.20. However, for each shock simulation, the same value of Ω is assumed for all nodes in the network. To analyze the results, we examine the avalanche sizes associated with different regions and layers. For each region, the number of avalanche sizes for a given value of Ω is 37, since the same region (node) is present in every layer of the network. Thus, regions represent transects of the multilayer network. For layers, the number of avalanche sizes is 115 since each layer consists of 115 geographic locations or nodes.

## Results

### Characterization of the multilayer network

The network has a high density, with 14,357,379 directed edges present in the network, of a total 18,105,025 possible edges (density ~ 0.79). The high density of this network indicates widespread dependencies amongst economic sectors and regions. While only 1.1% of the edges are intra-regional flows, ${S}_{ii}^{\alpha \beta}$, these flows represent 30% of all the intermediate demand. This finding mainly reflects the high reliance of regions on local services, such as retail, food and government services. Based on the in- and out-degree of nodes, the most central regions tend to be populous cities (Table 2), e.g., Chicago, Los Angeles and New York City. Later in this section, we re-examine the centrality of regions based on their tendency to generate large avalanche sizes.

**Table 2:** Regions with highest in- and out-degree, rounded to the nearest integer

Region	In-degree	Region	Out-degree
Chicago	142,815	Chicago	146,767
New York City	141,700	Los Angeles	140,640
Los Angeles	138,416	Saint Louis	140,331
Houston	137,006	Houston	139,699
Kansas City	136,788	Dallas	137,891
Saint Louis	136,723	Kansas City	137,179
Philadelphia	136,187	Atlanta	136,311
Atlanta	136,150	New York City	136,282
Dallas	134,550	Minneapolis	136,148
Minneapolis	134,368	Philadelphia	136,106

By separating the flows of the multilayer network into intralayer and interlayer flows, we find that 22% of the monetary flows occur in the same layer, ${S}_{ij}^{\alpha \alpha}$, while the remaining 78% of the monetary flows occur between two different layers, ${S}_{ij}^{\alpha \beta}$. These strong interdependencies between different economic sectors (layers) suggest that a shock to an individual economic sector has the potential to influence the network flows beyond what is captured through analyses of single-layer sectoral networks.

For each layer, the interlayer (Figure S1) and intralayer (Figure S2) in-degree and out-degree distribution is characterized by rapidly decaying tails. This is also the case for the interlayer (Figure S3) and intralayer (Figure S4) in-strength and out-strength distributions. A salient characteristic of the layers’ degree and strength distributions is that they tend to be peaked. This is due to the network being spatially constrained. The same has been observed in multiple other spatial networks (Barthélemy 2011). This means that one expects geographic location and distance to play an important role in the network.

Networks obtained from economic input-output data are generally characterized by a high connectivity (Acemoglu et al. 2016a; Blöchl et al. 2011). This is because any sector in the economy needs inputs from most other sectors for production. For instance, the manufactured food sector not only needs inputs from the agricultural sectors but also from industrial sectors, such as plastic and paper products, as well as services such as transportation and retail. Our multilayer network is characterized by a high connectivity, with each node being on average connected to roughly 2/3 of all the other network’s nodes. For such a high connectivity, one can expect shocks to potentially result in large avalanche sizes. It is, however, unclear how robust the network is to cascading failures and how robustness may vary across regions and sectors. We address these issues in the next subsections.

### Illustration of the network cascade model

**Fig. 2:**
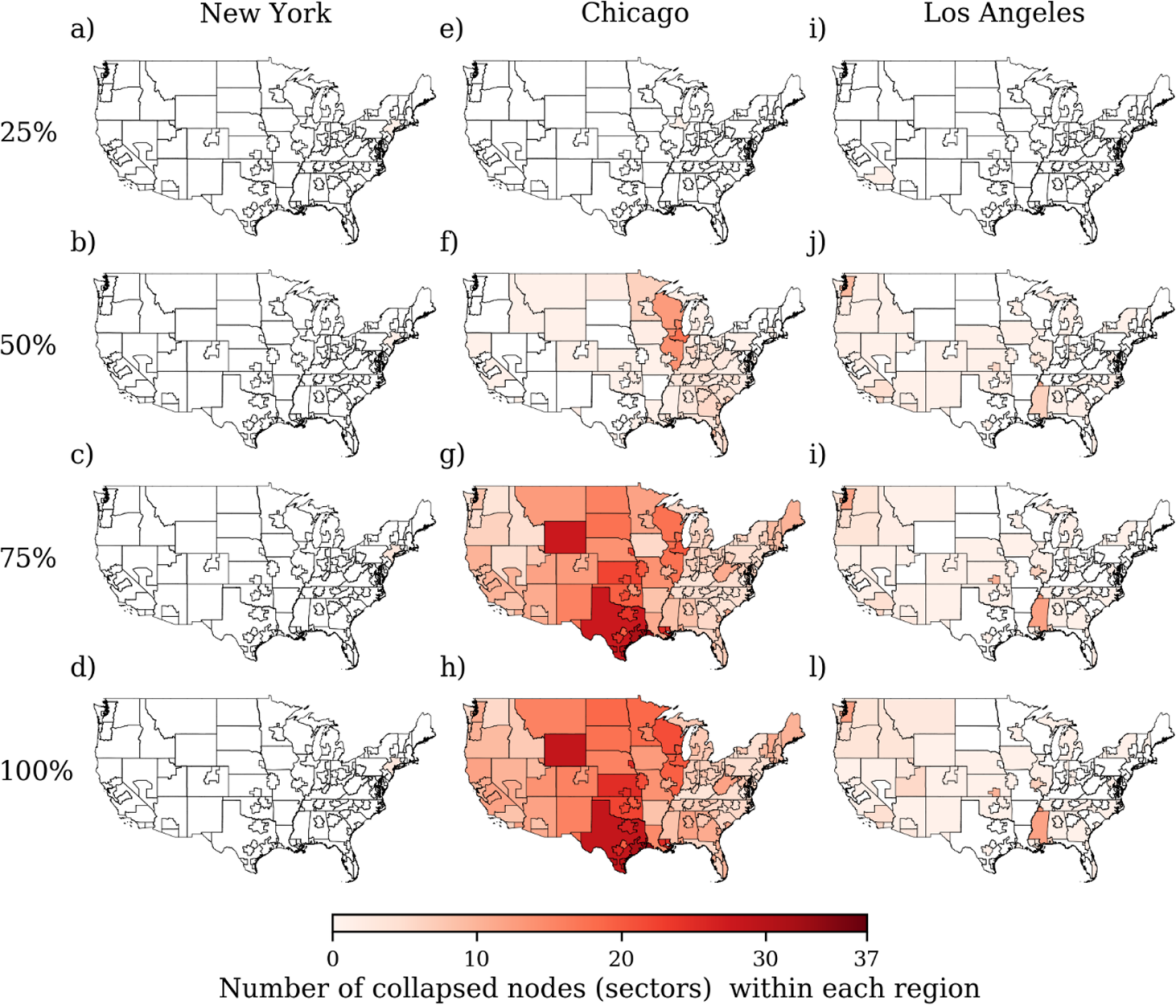
Spatial evolution of a shock initiated in the government sector of New York City (**a**-**d**), Chicago (**e**-**h**), and Los Angeles (**i**-**l**) for Ω = 0.06. The colors show the number of collapsed nodes within each geographical region at the specified level of propagation. The shock is shown at the 25 (**a**, **e**, **i**), 50 (**b**, **f**, **j**), 75 (**c**, **g**, **i**) and 100% (**d**, **h**, **l**) level of propagation relative to the total number of time steps or iterations required for the shock to be completely absorbed. The total number of time steps is 2, 25 and 12 for New York City, Chicago and Los Angeles, respectively. The avalanche size is 3, 1622 and 156 for New York City, Chicago and Los Angeles, respectively

We illustrate in Fig. 2 the spatial evolution of avalanches associated with shocks to the government sector in the three most populous cities in the United States for Ω = 0.06. Although the shock starts in the government sector of each of the three cities, the shock can propagate to other regions and sectors through the supply-chain connections. Our results show that, depending on the source node, the propagation of a shock can vary greatly in space (Fig. 2). For example, New York City is able to absorb the entire shock within its political boundary (Fig. 2a-d). In contrast, Chicago (Fig. 2e-h) and Los Angeles (Fig. 2i-l) are unable to buffer the shock and the shock propagates outside their political boundaries to affect different sectors in other regions. In the case of Chicago, the affected regions span the entire contiguous United States. The avalanche size is greater for Chicago than Los Angeles with 1622 and 156 collapsed nodes, respectively (Fig. 2).

For the three cities in Fig. 2, we include shock propagation maps for other values of Ω as Supporting Information. When Ω is relatively small (Ω = 0.02) (Figure S5), a shock to any of these three cities affects the majority of regions and sectors in the network, i.e. the propagation of a shock becomes less dependent on the source node. This is due to the relative density of the network, and suggests that efforts to avoid or mitigate cascading failures will require active maintenance and enhancement of network robustness.

Interestingly, shocks initially propagate following seemingly distinct inter-regional patterns. For instance, at the 50% level of propagation in Figure S5, the shock to New York City and Los Angeles is mostly contained within the northeastern and western area of the United States, respectively. This suggests the possibility of using inter-regional interactions to strengthen network robustness. The importance of inter-regional interactions in the structure of subnational economic flows has been highlighted before using single-layer networks for the United States (Garcia and Mejia 2019).

Another spatial tendency of the network is for regions in the central United States to experience the collapse of a greater number of nodes than other regions (Figure S6). This may be due to central regions serving as breadbaskets and manufacturing hubs for the rest of the country, particularly for meeting demand in populous cities of the East and West coasts. At a relatively higher value of Ω (Ω = 0.08) (Figure S7), the three cities are sufficiently robust to absorb the majority of the shock. That is, as Ω increases, the ability of shocks to propagate across regions and sectors declines.

Overall, Fig. 2 demonstrates that the cascade model applied to our multilayer network captures complex sectoral interdependencies that vary across space. This ability to resolve spatial interactions is an advantage of our multilayer approach compared to previous shock propagation analyses based on single-layer or national-level input-out data (Blöchl et al. 2011; Contreras and Fagiolo 2014; McNerney et al. 2013). This is particularly important for large countries like the United States that can exhibit pronounced internal variability in their economic interactions.

### Characterization of avalanche sizes across regions and layers

**Fig. 3:**
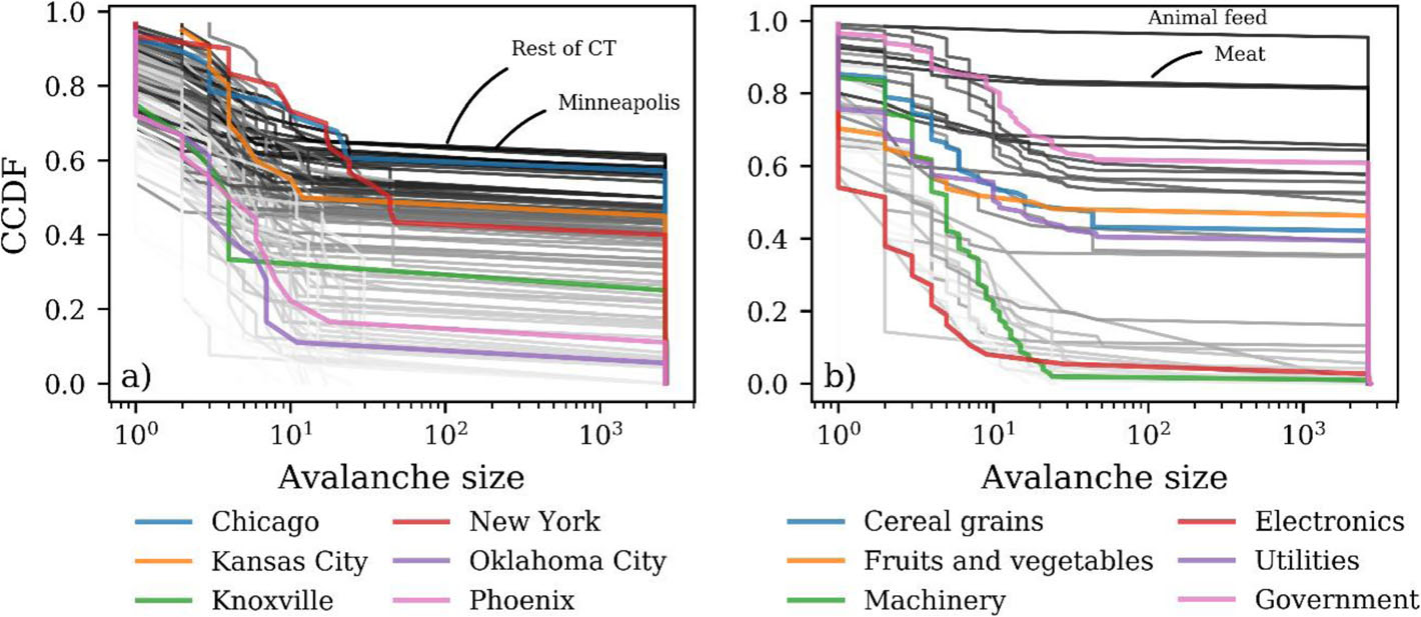
Complementary cumulative distribution function (ccdf) of avalanche sizes for the 115 regions (**a**) and 37 layers (**b**) in our multilayer network. The ccdfs are for Ω = 0.04

To characterize avalanche sizes across regions and layers, we use the complementary cumulative distribution function (ccdf) of the avalanche size. We show in Fig. 3 the ccdf of avalanche sizes for Ω = 0.04. The distribution of avalanches among regions is highly heterogeneous (Fig. 3a). For example, the probability of having an avalanche size greater than 20 collapsed nodes is 60% in Chicago, while the probability is only 10% for the same number of collapsed nodes in Oklahoma City (Fig. 3a). The probability of having large avalanche sizes (> 10^3^ collapsed nodes) varies from 7 to 55% among all regions (Fig. 3a). Likewise, heterogeneity is large for avalanches that originate within the same layer. The probability of having an avalanche size greater than 100 collapsed nodes is 1 and 62% for the machinery and government layers, respectively (Fig. 3b). For layers, the probability of large avalanches (> 10^3^ nodes) ranges from 1 to 95% (Fig. 3b).

The ccdf curves for some of the layers (Fig. 3b) have consistently higher probabilities than the ccdf curve for any of the regions (Fig. 3a). For instance, the top ccdf curve in Fig. 3b has a nearly constant probability of 1, indicating that the vast majority of nodes in that layer are characterized by relatively large avalanches. In contrast, the upper envelope in Fig. 3a shows a decline from a probability of 1 to 0.6. Thus, in the extreme case for regions, only ~ 60% of the nodes have a large avalanche size. This difference in the behavior of the top ccdf curves in Fig. 3a and b is partly because layers contain a greater number of nodes than regions, 115 versus 37, respectively. However, it also indicates that layers can have wide system-level repercussions that affect a large proportion of nodes in the multilayer network. For example, a shock to any of the nodes in the animal feed layer (top ccdf curve in Fig. 3b) is likely to cause the collapse of approximately half of all the multilayer nodes.

While the ccdfs in Fig. 3 are for Ω = 0.04, the ccdfs for other values of Ω are also characterized by high heterogeneity (Figures S8, S9, S10, S11 and S12). Overall, as the value of Ω declines, the probability of having large avalanches rises across regions and layers. For example, the maximum avalanche size for layers is 4050 collapsed nodes for Ω = 0.02 (Figure S8) and 33 collapsed nodes for Ω = 0.20 (Figure S12).

**Fig. 4:**
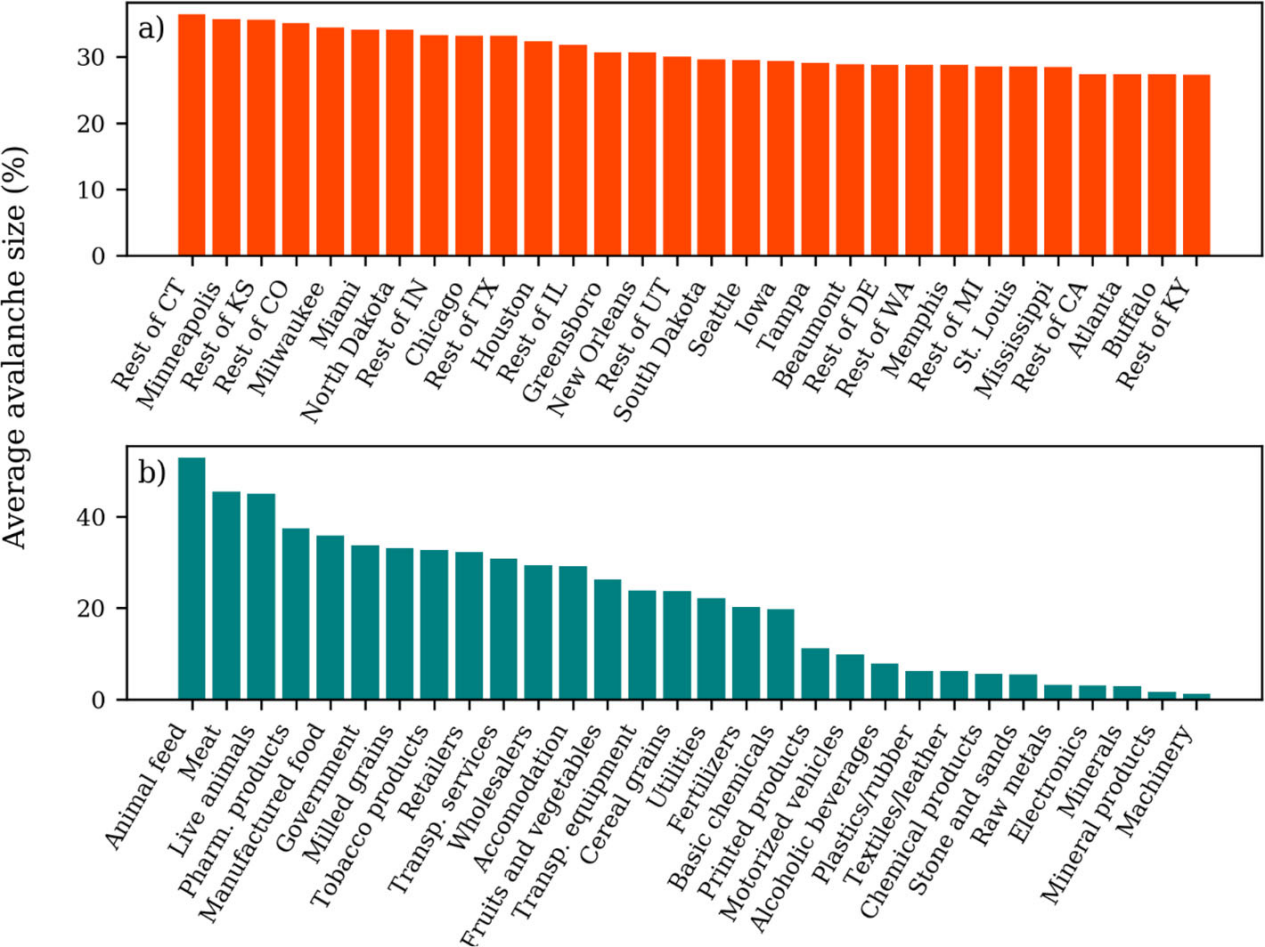
Average avalanche size for the top 30 regions (**a**) and layers (**b**) in the multilayer network (Ω = 0.04)

The regions with the highest average avalanche size are varied (Fig. 4a). For example, the 30 regions with the highest average include breadbasket regions (e.g., rest of California, Iowa, rest of Kansas and rest of Texas), large cities (e.g., Chicago, Houston and Miami), manufacturing hubs (e.g., Milwaukee, rest of Indiana and rest of Mississippi) (US Cluster Mapping 2019) and trade hubs (e.g., Buffalo, Memphis and New Orleans) (Peryea 2018) (Fig. 4a). The layers with the highest average are dominated by food-related and service sectors (Fig. 4b). In fact, the top 17 layers with the highest average consist of food-related and service sectors, with the only exceptions being pharmaceutical products, tobacco products and transportation equipment (Fig. 4b). The results are similar for other values of Ω.

Some of the layers with the highest average avalanche size (Fig. 4b) are also sectors that have been severely disrupted by the COVID-19 pandemic. The pandemic has significantly stressed supply chains in the United States but some sectors have proven to be more fragile than others, such as the meat (Corkery and Yaffe-Bellany 2020) and produce (Reiley 2020) sectors. It is encouraging that our multilayer network of supply chains, along with the network cascade model, are able to distinguish these same sectors as susceptible to the generation of large cascade events.

### Response of the supply chains to shocks

**Fig. 5:**
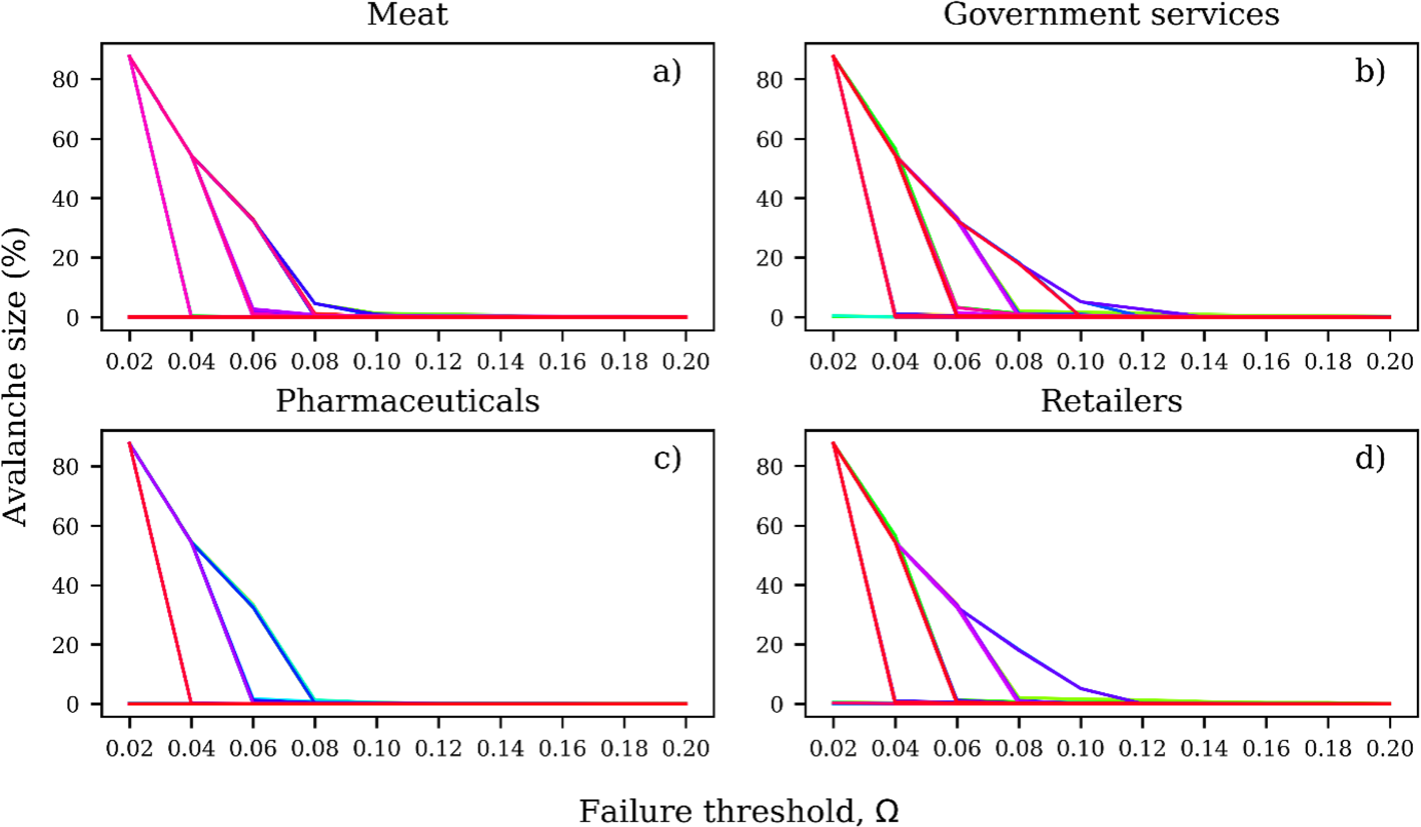
Avalanche sizes for each node in the meat (**a**), government services (**b**), pharmaceuticals (**c**) and retailers layer (**d**) versus the failure threshold Ω. Each curve represents the avalanche size-failure threshold relationship for a node in the layer

By examining how the avalanche size for each node varies with respect to the failure threshold Ω, we explore the response of the supply chains to shocks. For each node in the multilayer network, the avalanche size exhibits a threshold-like behavior with respect to Ω (Fig. 5). We show in Fig. 5 the avalanche sizes associated with each node in four different layers against the value of Ω. For the meat layer (Fig. 5a), the avalanche size is small or negligible for Ω ≥ 0.08 but rises relatively quickly for Ω < 0.08, indicating that below this level of Ω shocks lead to catastrophic failures. The behavior is similar for nodes belonging to other layers (Fig. 5b-d). For example, the avalanche size for pharmaceutical products also shows a sudden increase for Ω < 0.08 (Fig. 5c). Likewise for the government and retailers layer (Fig. 5b and d), although in these layers the avalanche size starts increasing at values of Ω < 0.10 and the increase is somewhat more gradual.

Nodes tend to generate the same or very similar avalanche sizes (Fig. 5). For instance, the curves associated with individual nodes in Fig. 5 tend to converge along the upper envelope curve representing the maximum avalanche size. This is because large avalanches tend to exploit similar failure paths as the cascade event progresses over time, suggesting that some nodes in the network might be more exposed than others to avalanches originating in other nodes. This is further explored in the next subsection of the results, where we use the exposure to shocks to better understand the relative role of nodes in the propagation of shocks.

**Fig. 6:**
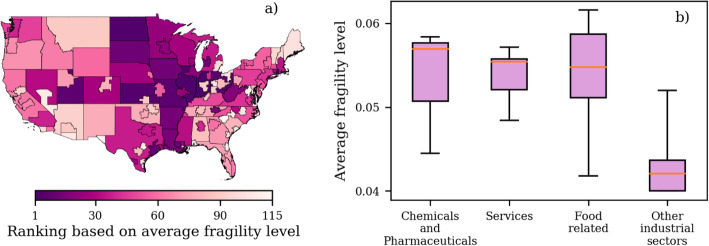
Ranking of all the regions from high to low (1–115) based on the average fragility level Ω_*c*_ of the nodes belonging to each region (**a**). Groups of layers with high and low fragility based on the average fragility level Ω_*c*_ of the nodes belonging to each layer (**b**). The layers in the food-related group include cereal grains, milled grains, fruits and vegetables, meat, animal feed, live animals, manufactured food, and fertilizers. The layers in the chemicals and pharmaceuticals group include basic chemicals, chemical products and pharmaceutical products. The layers in the services group include government, food services (e.g., restaurants), wholesalers, retailers, transportation services, government and utilities. The group labeled other industrial sectors includes the rest of the layers that are not part of the high-fragility groups. In the box-and-whisker plots, the box represents the first and third quartile, the red line is the median and the whiskers show the range of the values

Since the supply chains reveal a threshold-like response to shocks, the critical value of Ω below which large cascades occur can be used to quantify the overall fragility level of a node. This critical value is referred to as Ω_*c*_. We utilize Ω_*c*_ to rank the overall fragility of regions. Based on Ω_*c*_, the regions with the highest fragility rank tend to be located in the central United States (Fig. 6a), including regions such as Arkansas, rest of Illinois, rest of Indiana, Iowa, rest of Kansas, and rest of Ohio, among others. These regions are all major producers of food-related products. The map in Fig. 6a indicates that shocks originating within the local economies of high-fragility regions are able to propagate more easily than shocks in low-fragility regions to generate large avalanche sizes in the network. For example, the average avalanche size is 509 and 155 for the high- and low-fragility region of Iowa and Los Angeles, respectively, for Ω = 0.06_._

We also use Ω_*c*_ to distinguish between high- and low-fragility layers. Based on Ω_*c*_, three different groups of layers are identified as having on average a high fragility: chemicals and pharmaceuticals, services and food-related (Fig. 6b). In Fig. 6b, the low-fragility group includes all the other layers that are not part of the high-fragility groups. Although the range of the low-fragility group is wide, the interquartile range shows a marked difference between the values of Ω_c_ for the low- and high-fragility layers (Fig. 6b). The median Ω_c_ is ~ 0.043 and ~ 0.055–0.057 for the low- and high-fragility layers, respectively. A higher median indicates layers that are more likely to result in large avalanche sizes.

However, the high-fragility layers alone do not determine the propensity of regions to generate large avalanche sizes. For example, services are locally important to all regions in our dataset since each region depends on its own internal supply of services to function. In addition, service layers show a more consistent high-fragility level. The interquartile range and the range of values is narrower for the services group (Fig. 6b). Even though each region contains nodes that belong to the same high-fragility service layers, this does not imply that all regions have a comparable fragility (Fig. 6a). It indicates instead that the connectivity of a region matters for the generation of large avalanche sizes. That is, for regions to have a high fragility level, their nodes need to be connected to neighbors and, more generally, paths that allow the propagation of shocks.

### Exposure to shocks and fragility risk

We use the exposure of a node to shocks to explore the role of network connectivity in the propagation of shocks. A node is more exposed to shocks when is more frequently affected by avalanches originating in other nodes in the network. Exposure serves to measure the relative importance of a node in the transmission of shocks. Measuring exposure thus requires the selection of an appropriate value of Ω. We use the avalanche size immediately below the fragility level Ω_c_ because this allows us to be consistent in our selection of avalanches while retaining more information. Note that if the value of Ω is too large or small, less information is available to distinguish the exposure of nodes. For instance, a small value of Ω results in large avalanches that affect most nodes in the network, implying a similar exposure for any node. We do not use a node-centrality metric, such as the node in- or out-strength, because they are based on the local connectivity of a node.

**Fig. 7:**
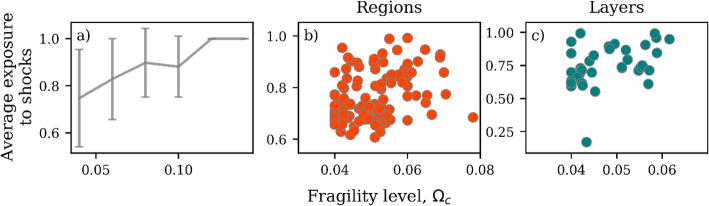
Average exposure of nodes versus the fragility level (**a**). The error bars indicate ± 1 standard deviation. Exposure of regions (**b**) and layers (**c**) to shocks versus the fragility level

We find that the average exposure of nodes tends to increase with the fragility level (Fig. 7a). Nodes with higher fragility levels are also more likely to be affected by shocks originating in other nodes. This means that the fragility level of a node partially depends on the fragility level of other nodes through the connectivity of the network. The range of variability, however, is wide in Fig. 7a, suggesting that node-level interventions are likely to be ineffective for this network. That is, changing the fragility level of a single node, e.g., by increasing its buffering capacity, will not necessarily improve its exposure to shock. This situation is further complicated by the fact that domestic supply chains are largely self-organized, which in general makes system-level interventions challenging to implement.

When nodes are aggregated into regions (Fig. 7b) or layers (Fig. 7b), the average exposure does not depend on the fragility level. This indicates the fragility risk is heterogeneous across regions and layers. The risk is higher for regions and layers with both a high exposure and high fragility level. Some of the regions with high risk are Baton Rouge, Houston and the rest of Kansas, while some of the high-risk layers are animal feed, basic chemicals and meat. These are regions and layers where the collapse of a single node is more likely to result in the collapse of the entire region or layer. The regions and layers with reduced risk have a low exposure and low fragility level. For example, San Diego and Washington DC are low-risk regions while furniture and newsprint/paper are low-risk layers.

Our results in Fig. 7b-c show that depending on the region or layer different strategies might be necessary to strengthen the supply-chain network. For example, regional-level interventions might suffice for regions with a relatively low exposure to shocks. However, in the case of high-risk regions or layers, an effective intervention is likely to involve both increasing the buffering capacity and reducing exposure. To address the latter, it becomes necessary to explore broader system-level solutions. It thus seems that to make the supply-chain network more robust to cascade events requires solutions at multiple levels of network aggregation.

## Discussion

### Implications

Overall, our results indicate that intranational supply-chain shocks can result in large cascading events due to the high connectivity of the network, the fragility level of nodes and the tendency of high-fragility nodes to interact with each other. Following from this, the main implication of our analysis is that there are groups of supply-chain linkages that facilitate the propagation of shocks.

The ability of sectoral-level shocks to generate cascading events was initially downplayed by economists (Lucas 1977). Recently, however, this view has been challenged by combining network-based approaches with macroeconomic models and empirical input-output networks. For example, Acemoglu et al. (2012) demonstrated that sectoral interdependencies can amplify sectoral-level shocks resulting in measureable macroeconomic change. Furthermore, Acemoglu et al. (2016a) found that the geographic location of sectors may act as another mechanism in the amplification of shocks. Although the latter finding was assessed using input-output data, they did not use spatially explicit data as we do in this study. The use of spatially explicit input-output data enables us here to jointly analyze the network effects associated with sectoral interdependencies and geographic location in the transmission of shocks. By doing so, our approach moves beyond previous analyses.

The fragility results provide an explanation for the emergence of cascading events. We find that services are a high-fragility group. Services are essential to the internal functioning of any region in our network, since services are locally provided. This means that the high fragility of services has a similar effect on any node in the network, and therefore services alone do not explain in our results the different fragility levels of regions. Services are also characterized by strong interactions (high strengths), this is partly what makes them more fragile than other layers. The high strength of services creates strong couplings between layers, within the same region, facilitating the transmission of a shock within a region. If that region’s nonservice sectors have also a high fragility (e.g., the region is a main producer of food products), the shock is likely to propagate outside the region creating a cascading event. This seems to be the case for regions in the central United States, given that these regions specialize in food production and are identified by our analysis as the most fragile.

Although our results are for the United States, our multilayer network approach is general and could be applied to other countries, assuming the necessary data are available. Moreover, the input-output network of the United States shares key features in common with networks in several other developed countries, such as the ranking of sectors’ centrality (Blöchl et al. 2011), which could make our results relevant to those countries.

### Limitations

Several limitations exist within this study that could be further refined for future research. The MRIO dataset used to build the multilayer network is for 2012. This year is used since it is the latest year of publicly available data. MRIO data, however, are known to only vary slowly over time (Acemoglu et al. 2016a; Blöchl et al. 2011), which makes using 2012 data less constraining. For example, a comparison between domestic trade flows between 2012 and 2018 shows that their correlation is high. The linear correlation is > 0.98 for any of the nonservice sectors in the MRIO dataset. Domestic trade flows are one of the main input data used to calibrate the MRIO dataset. Based on this, we do not expect to observe drastic differences between the 2012 MRIO dataset and a dataset representing conditions prior to the pandemic.

The results within this network analysis are based on the value of a commodity group through monetary units of analysis (USD). While appropriate for this study, monetary units serve as a coarse estimate of value and would likely not be the most important factor when designing policy interventions. For example, in times of widespread economic disruption, conditions relating to employment or jobs by sector may be of principle concern. Other units of measure such as environmental extensions in water or carbon have provided useful understandings of sustainability implications for supply-chain networks (Garcia and Mejia 2019; Garcia et al. 2020b).

Another challenge stems from assuming a value of Ω and applying the same Ω to all nodes when propagating a shock. Future studies could use empirical estimates for Ω, which could allow Ω to vary from node to node. Finally, this model assumes that once a node collapses, it does not recover during the cascade event. In the future, allowing nodes to recover and collapse again during a cascade event may provide varying results.

### Future research: fragility, critical infrastructure, security, and ethics

Future work might look to fragility as we have measured it here for deeper study of unequal distribution of risk. Understanding fragility of individual sectors and regions (Figs. 5 and 6) as an indicator of risk allows ethics-driven inquiry into equity and fairness underlying interdependent critical infrastructure. It provides, we argue, a direction for data-driven policies aimed at providing support to vulnerable populations in times of disruption. To prompt discussion on future research directions unpacking fragility and supply chains risk, we briefly elaborate on two thematic foci, specifically critical infrastructure framing and the ongoing COVID-19 pandemic, within this discussion section.

Critical infrastructure (CI) security has been sustained as a priority under multiple administrations in the United States (TWH EO 2017; TWH PPD 2013). CI encompasses the 16 sectors whose physical assets or virtual systems are so critical to the United States that their disruption would have devastating effects on economic security, public health, or safety (TWH EO 2017; TWH PPD 2013). Two of the three groups found through our study results directly overlap with two of these CI sectors. Our layers of food-related and chemical/pharmaceutical products mirror the CI sectors of Food and Agriculture and Chemicals Sector and both rely on several of the other CI sectors. Furthermore, the services group, which includes the government, wholesale, retailers, transportation and utilities layers, relates to several more CI. Our work has simulated disruptions throughout supply chains in the United States where collapsing avalanches jeopardize the provisioning of goods and services in spatially varied ways. Several scholars articulate that if we hope to support basic human needs such as provisioning of food and water understanding prioritization of risk, responsibility and security around CI will be of critical importance (Clark et al. 2018; Garschagen and Sandholz 2018).

In addition to CI broadly, the COVID-19 pandemic has shown that the unequal distribution of risk and impact can exacerbate the negative consequences of cascading failures. The pandemic has impacted US and global communities with unprecedented reach, leaving virtually no one unaffected. However, already, just months into the crisis, outsized impacts on vulnerable Americans are becoming clear. Uninsured and under-insured individuals are more likely to have underlying conditions which make them disproportionately susceptible to serious cases of coronavirus (Akpan 2020; Rutkin 2020). This group overlaps with the subset of low-income essential workers still required to go into work every day, a disproportionate share of whom are racial and ethnic minorities (Quinn et al. 2011). Food insecurity has heightened as schools close, food banks suspend operations and millions are impacted by layoffs (Aridi 2020; WFP USA 2020). From the perspective of supply chain disruptions, we have witnessed a bidding war amongst state and local governments over sought-after medical equipment (Estes 2020). Hospitals in rural communities with fewer resources generally rely on transferring their patients to larger, metropolitan hospitals. But as those hospitals reach capacity, those safety nets may fall away (Schumaker and Parks 2020).

In sum, as future research, we highlight the need of exploring fragility, not only by studying large sections or regions as we have done within this paper, but also through a deeper study of unequal distribution of impact. This will be critical to elucidate data-driven policies aimed at building resilience across populations in times of disruption.

Another area for future research is to consider the effects of international flows and different socioeconomic drivers on the supply chains’ fragility. International flows, imports and exports, can amplify or dampen the propagation of shocks, which could influence the nodes’ fragility. An econometric model could be used to identify the different drivers (e.g., economic growth, employment, demographics, industry specialization, supply-chain diversity, product complexity, etc.) of the nodes’ fragility, which could also potentially serve to anticipate cascading events. Another option would be to use a network-based macroeconomic model (Carvalho and Tahbaz-Salehi 2019) to explore the effects of shocks on macroeconomic change.

## Conclusions

In this study, we build a multilayer network to represent supply chains in the United States. The network’s links and weights are determined using a multiregional input-output dataset. Together with a cascade model, the multilayer network is utilized to explore the propagation of economic shocks along intranational supply chains.

Based on our simulation results, we find that the impact of a shock, measured using the avalanche size, varies widely depending on the source node, i.e. the geographic region and economic sector of origin of the shock. By varying the failure threshold of a node, the response of supply chains to shocks reveals a threshold-like behavior. Below a certain value of the failure threshold, the avalanche size tends to grow relatively quickly for any node in the network. We interpret such threshold as the fragility level of a node since it is indicative of a node’s tendency to generate large avalanche sizes.

Using the fragility level to rank regions, we find that the most fragile regions tend to be located in the central United States. These are regions that specialize in the production and manufacturing of food-related products. This result is corroborated by examining the fragility level of individual network layers. The most fragile layers fall into three groups: food-related products, chemical and pharmaceutical products, and services. Relevantly, these are all sectors that have been disrupted by the COVID-19 pandemic in the United States. Disruptions to the service sectors are expected from the pandemic because of reduced human mobility due to stay-at-home orders. The disruptions to the other two groups (food-related and chemical/pharmaceutical products) are less direct and suggest that supply-chain fragility might indeed be a consequential issue within the United States.

By jointly examining a node’s fragility level and exposure to shocks, we assess a node’s fragility risk. Although there is a tendency in the network for high-fragility nodes to be more exposed to shocks, the fragility risk is heterogeneous across regions and sectors. Based on this result, we suggest that to mitigate cascading events in the network different strategies might be necessary depending on the fragility risk. Further, we believe our results have practical implications. In the discussion, we have emphasized ethical and infrastructural implications in the context of unequal distribution of risk and the COVID-19 pandemic. Other areas of application could involve the design and testing of interventions aimed at enhancing network robustness.

## Supplementary information

**Additional file 1. Supporting Information.**

## Data Availability

The dataset is available by request through the HydroShare repository of The Consortium of Universities for the Advancement of Hydrologic Science at http://www.hydroshare.org/resource/8059b9c24d2e4f1d8bd28d04758b007a.

## Abbreviations

CCDF: Complementary cumulative distribution function
CI: Critical infrastructure
COVID-19: Coronavirus Disease 2019
MRIO: Multiregional input-output
USD: United States dollar
