# Supplementary material for: Fragility of a multilayer network of intranational supply chains
Source: Appl Netw Sci. 2020 Sep 23;5(1):71. doi: 10.1007/s41109-020-00310-1 (PMC7509503; doi:10.1007/s41109-020-00310-1)
Supplement: Supplementary file 1 — Additional file 1. Supporting Information. [file 41109_2020_310_MOESM1_ESM.docx]

**Supporting Information**

**Fragility of a multilayer network of intranational supply chains**

Michael Gomez^1^, Susana Garcia^1,2^, Sarah Rajtmajer^3,4^, Caitlin Grady^1,4^, and Alfonso Mejia^1*^

^1^Department of Civil and Environmental Engineering, The Pennsylvania State

University, USA

^2^Postdoctoral Research Associate, Energy and Transportation Science Division, Oak Ridge National Laboratory, USA

^3^College of Information Sciences and Technology, The Pennsylvania State University, USA

^4^The Rock Ethics Institute, The Pennsylvania State University, USA

^*^Corresponding Author: aim127@psu.edu


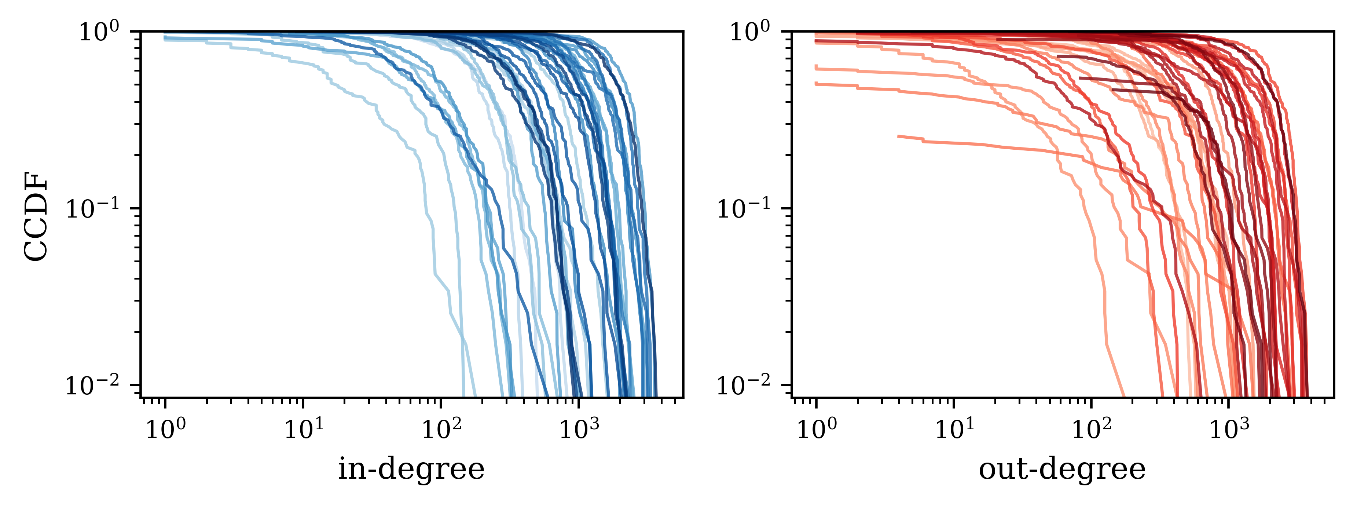


**
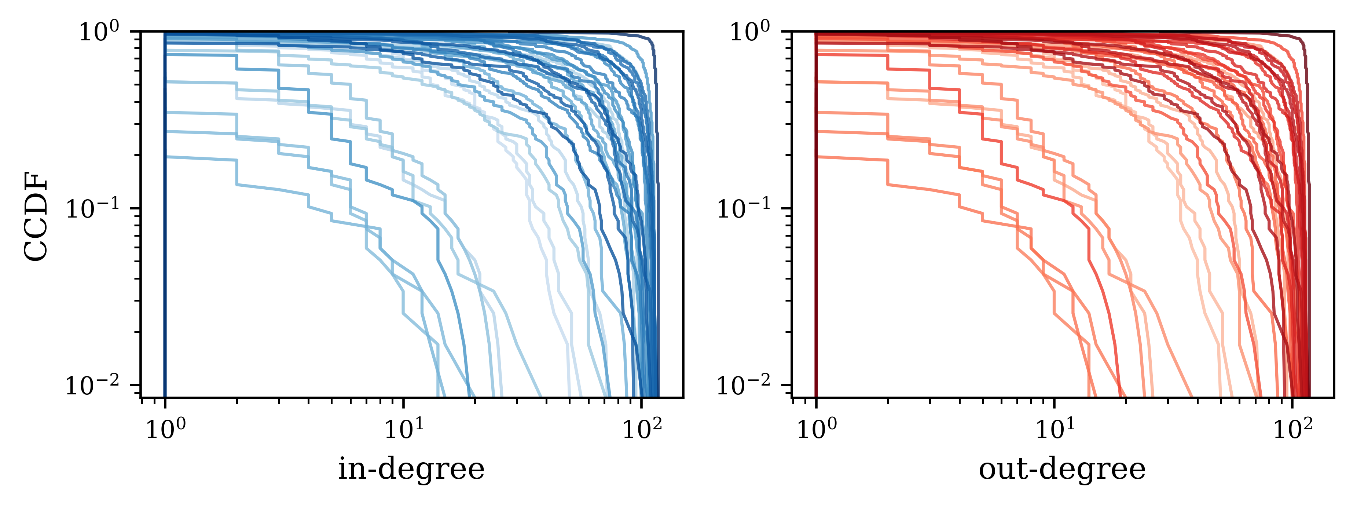
Fig. S1** Complementary cumulative distribution function of the interlayer in-degree (**a**) and out-degree (**b**) for each layer.

**Fig. S2** Complementary cumulative distribution function of the intralayer in-degree (**a**) and out-degree (**b**) for each layer.


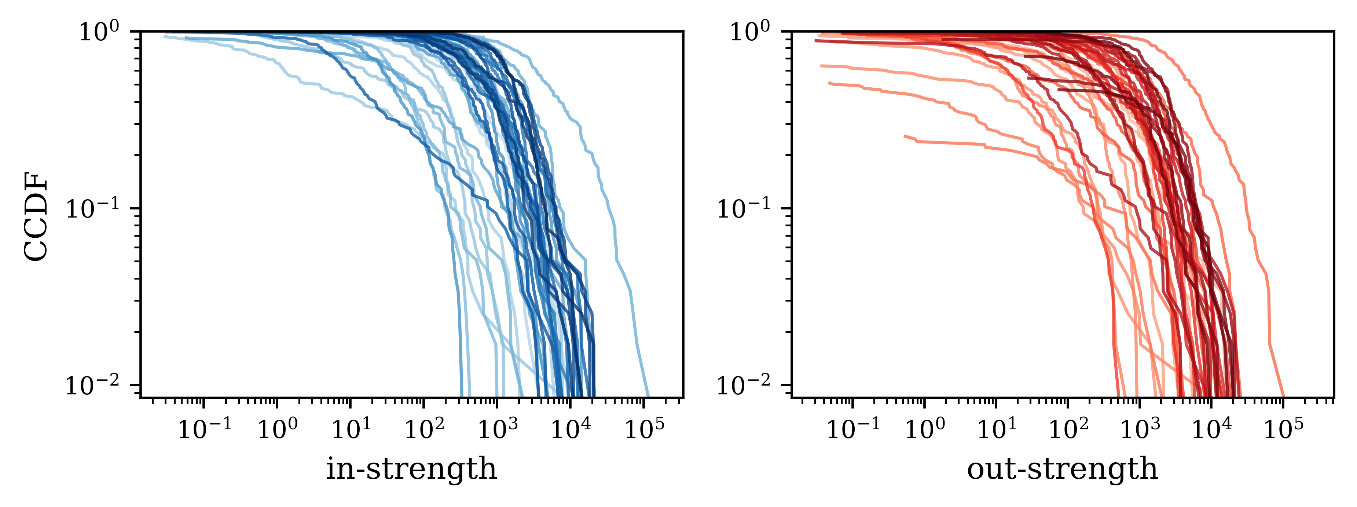
 **Fig. S3** Complementary cumulative distribution function of the interlayer in-strength (**a**) and out-strength (**b**) for each layer.


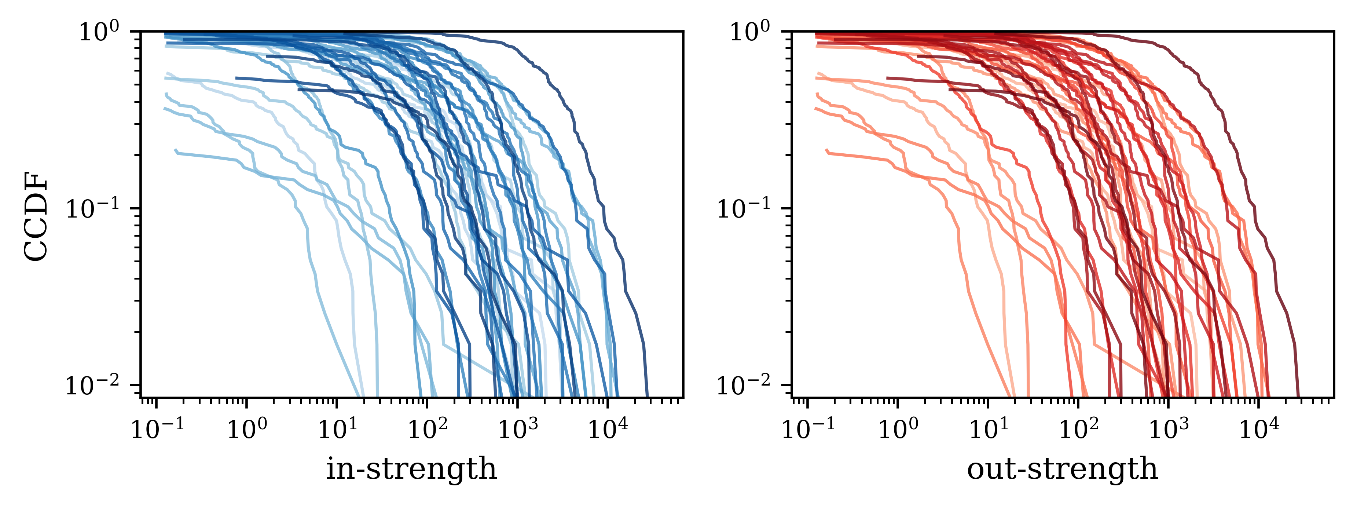


**Fig. S4** Complementary cumulative distribution function of the intralayer in-strength (**a**) and out-strength (**b**) for each layer.

**
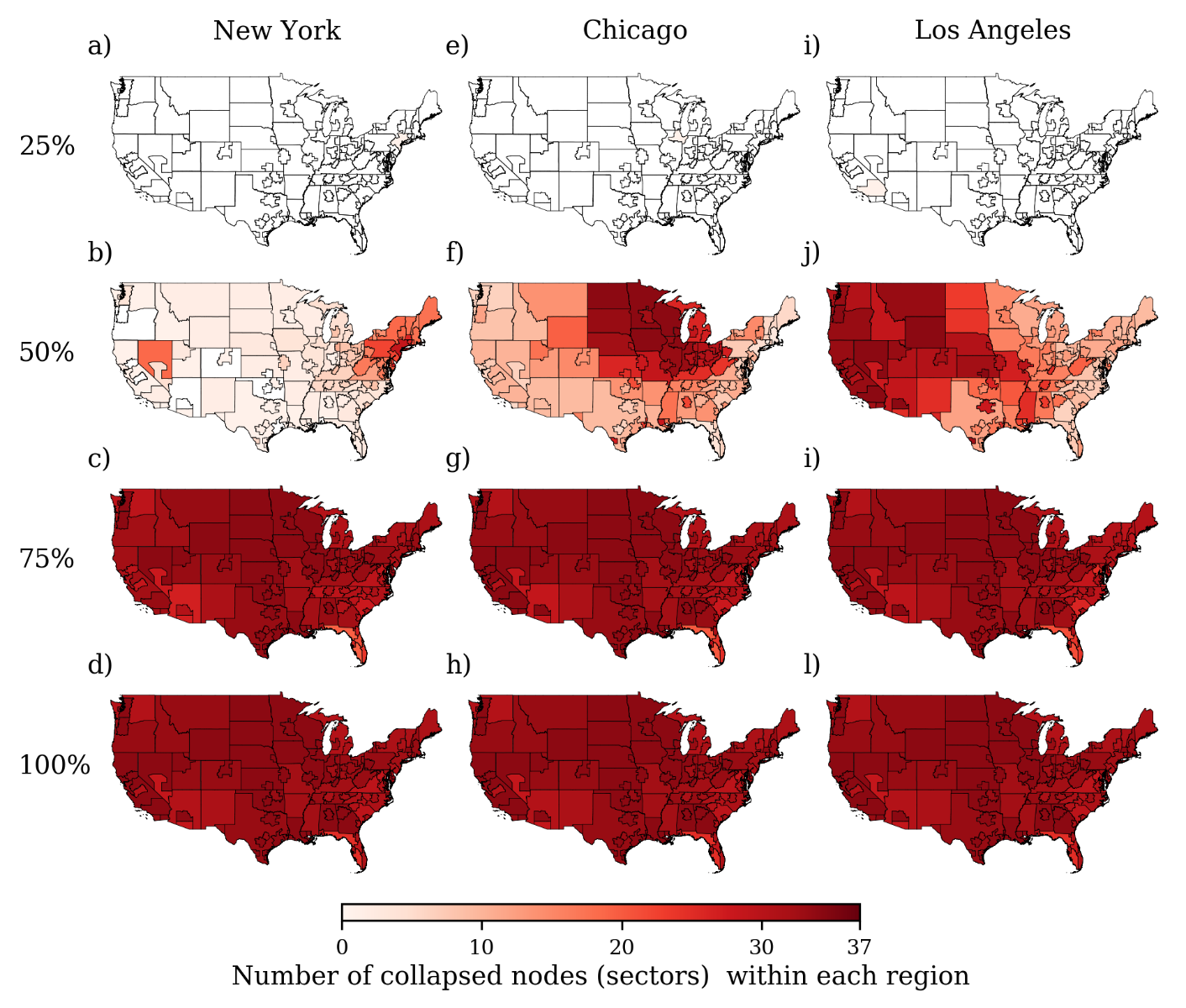
**

**Fig. S5** Spatial evolution of a shock initiated in the government sector of New York City (**a-d**), Chicago (**e-h**), and Los Angeles (**i-l**) for Ω=0.02. The shock is shown at the 25, 50, 75 and 100% level of propagation relative to the total number of time steps or iterations required for the shock to be completely absorbed.

**
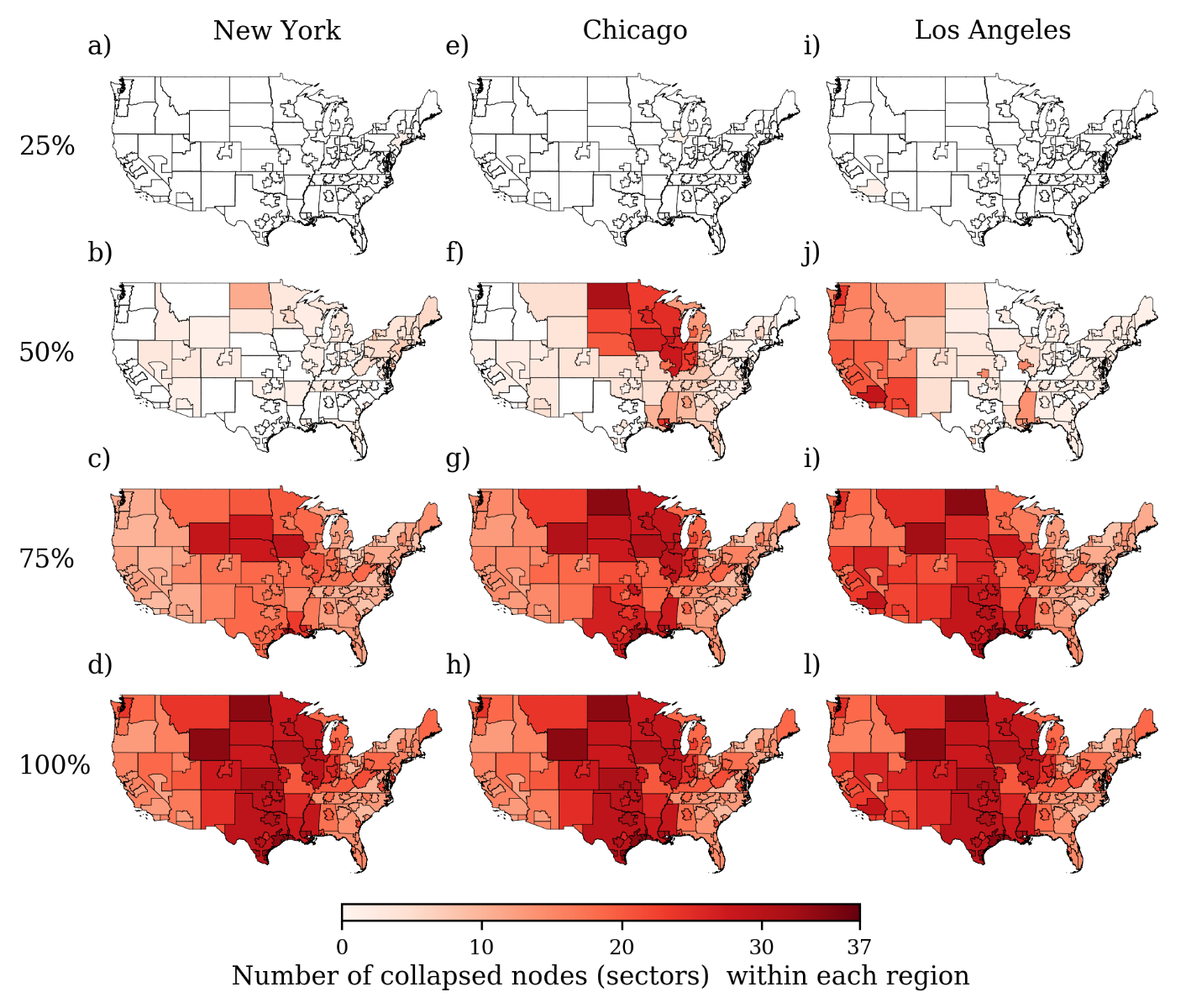
**

**Fig. S6** Spatial evolution of a shock initiated in the government sector of New York City (**a-d**), Chicago (**e-h**), and Los Angeles (**i-l**) for Ω=0.04. The shock is shown at the 25, 50, 75 and 100% level of propagation relative to the total number of time steps or iterations required for the shock to be completely absorbed.

**
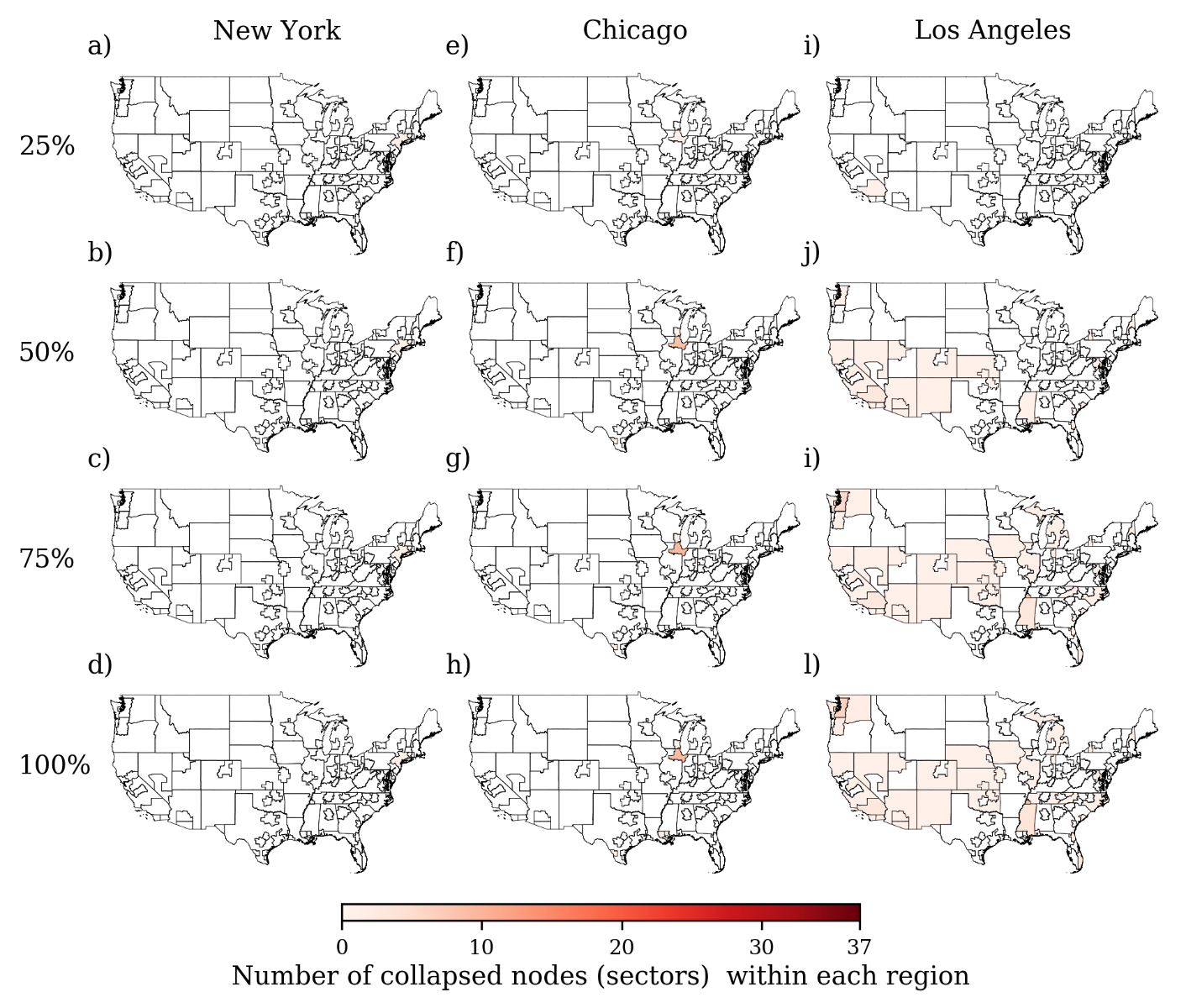
**

**Fig. S7** Spatial evolution of a shock initiated in the government sector of New York City (**a-d**), Chicago (**e-h**), and Los Angeles (**i-l**) for Ω=0.08. The shock is shown at the 25, 50, 75 and 100% level of propagation relative to the total number of time steps or iterations required for the shock to be completely absorbed.


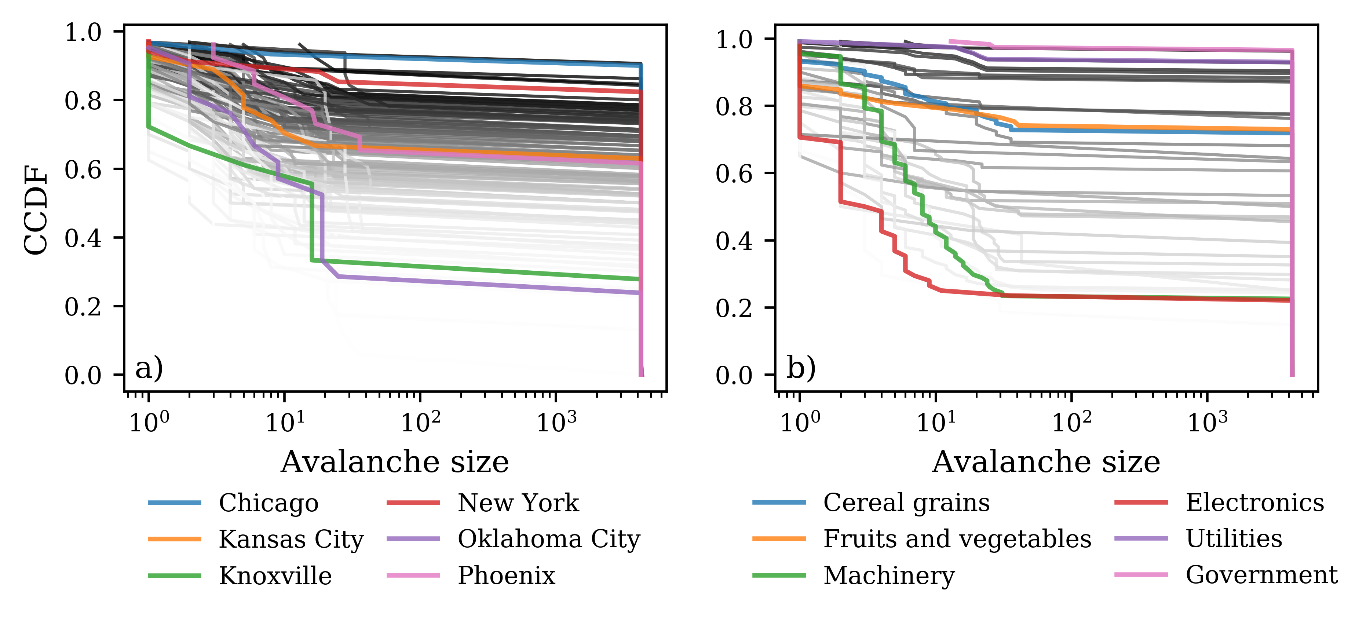


**Fig. S8** Complementary cumulative distribution function (ccdf) of avalanche sizes for the 115 regions (**a**) and 37 layers (**b**) in our multilayer network. The ccdfs are for Ω=0.02.


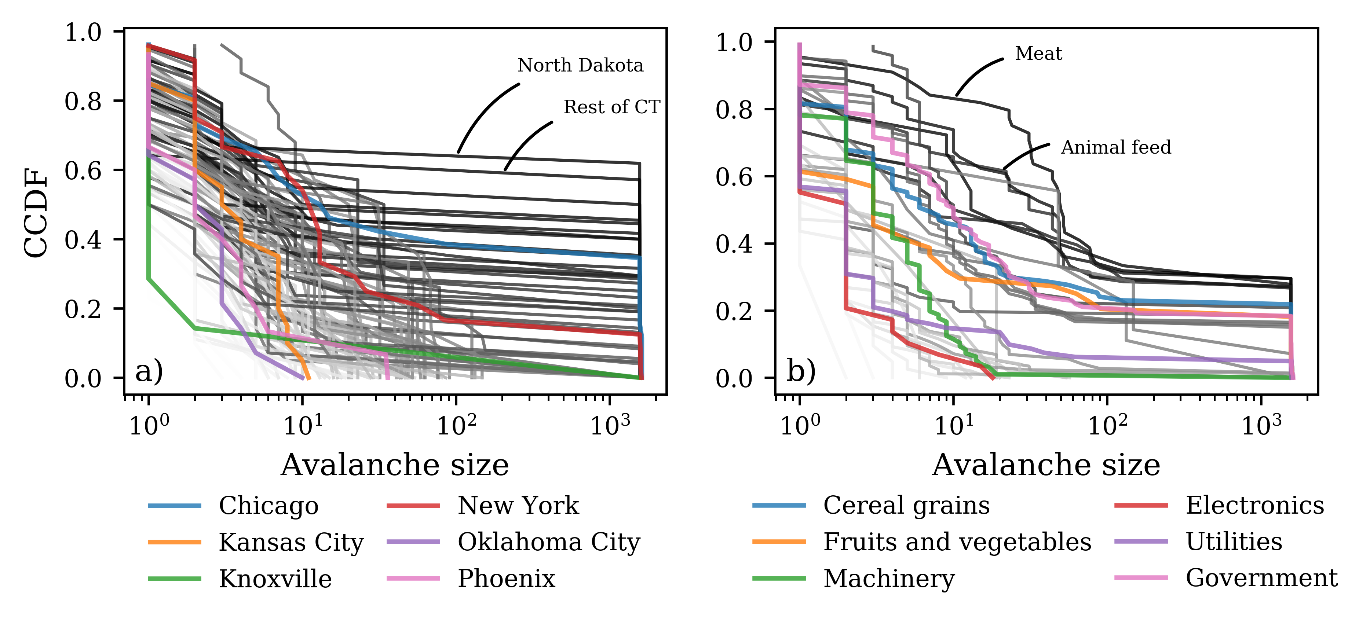


**Fig. S9** Complementary cumulative distribution function (ccdf) of avalanche sizes for the 115 regions (**a**) and 37 layers (**b**) in our multilayer network. The ccdfs are for Ω=0.06.

**
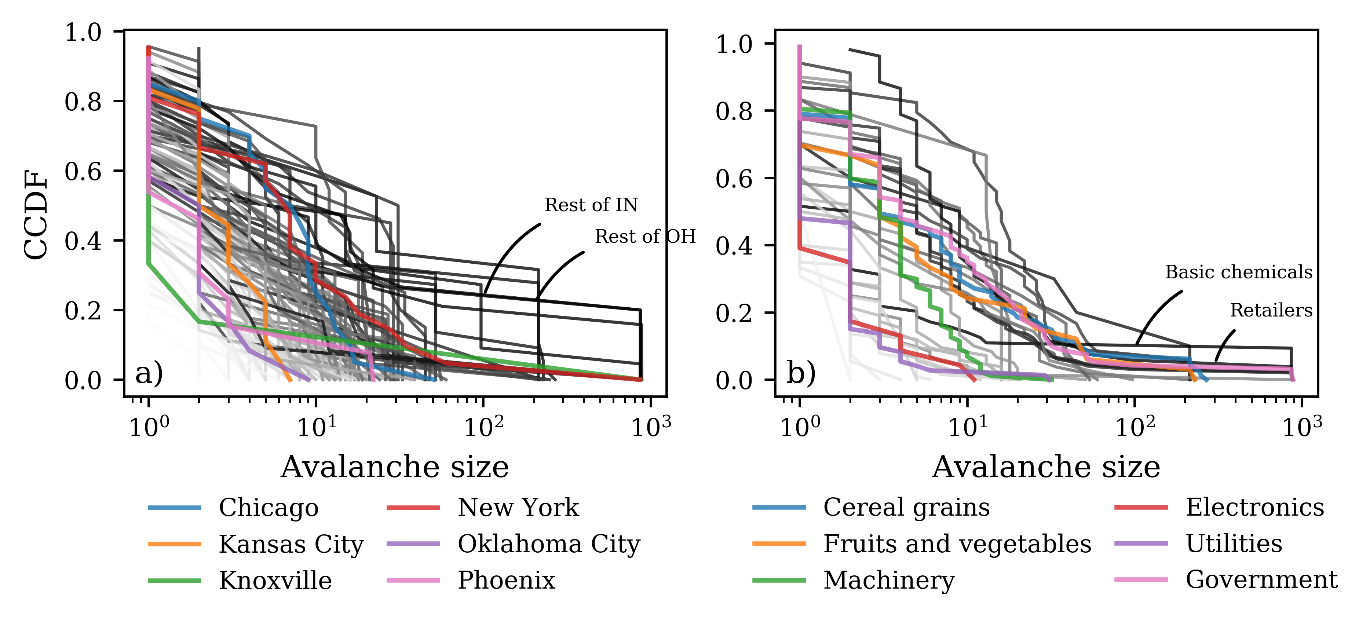
Fig. S10** Complementary cumulative distribution function (ccdf) of avalanche sizes for the 115 regions (**a**) and 37 layers (**b**) in our multilayer network. The ccdfs are for Ω=0.08.

**
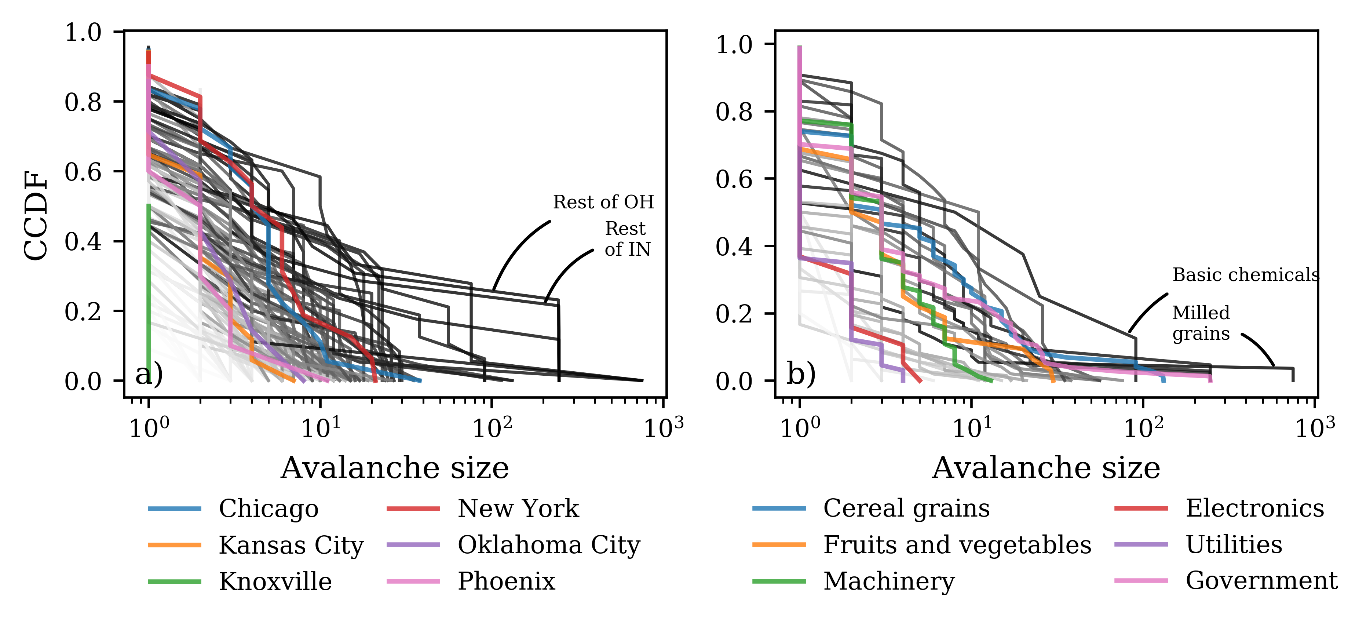
**

**Fig. S11** Complementary cumulative distribution function (ccdf) of avalanche sizes for the 115 regions (**a**) and 37 layers (**b**) in our multilayer network. The ccdfs are for Ω=0.10.

**
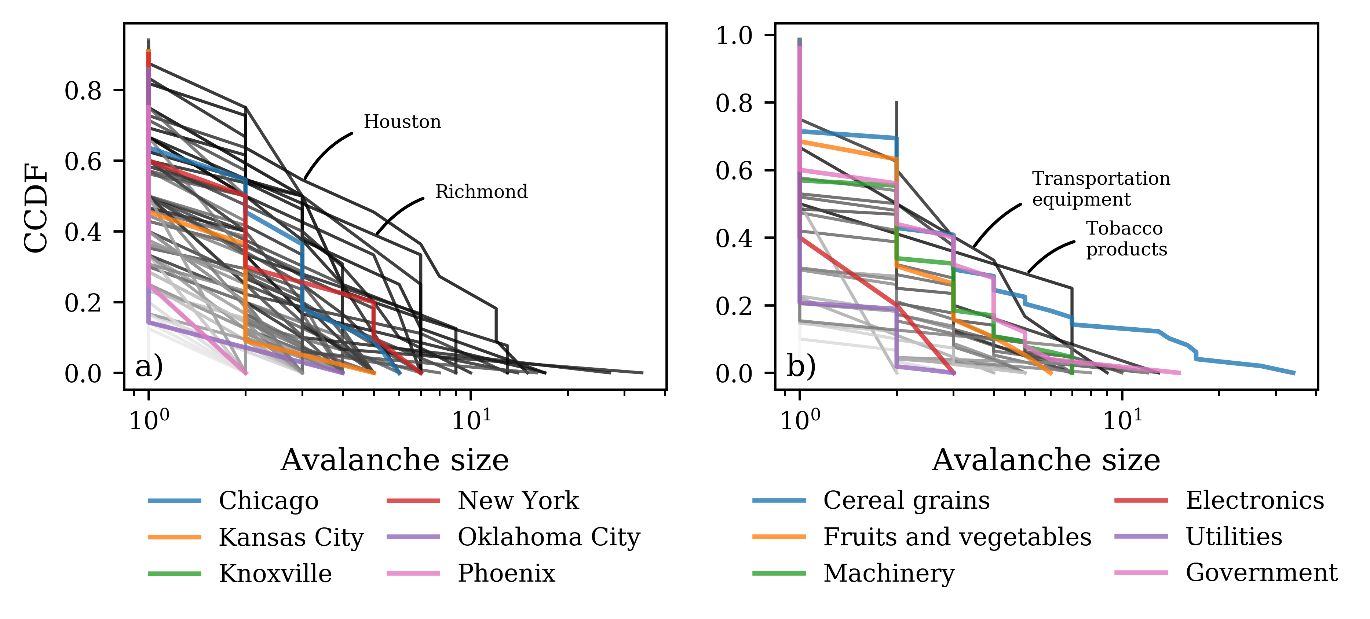
Fig. S12** Complementary cumulative distribution function (ccdf) of avalanche sizes for the 115 regions (**a**) and 37 layers (**b**) in our multilayer network. The ccdfs are for Ω=0.20.
